# Renal Net Acid Excretion During Growth and eGFR, Creatinine Clearance, and Albuminuria in Young Adulthood

**DOI:** 10.1016/j.xkme.2025.101195

**Published:** 2025-12-02

**Authors:** Thomas Remer, Seyedeh-Masomeh Derakhshandeh-Rishehri, Yifan Hua, Luciana Peixoto Franco, Hermann Kalhoff, Stefan A. Wudy

**Affiliations:** 1 DONALD Study Center, Nutritional Epidemiology, Institute of Nutrition and Food Science, University of Bonn, Dortmund, Germany; 2 Research Department of Child Nutrition, St. Josef-Hospital, University Hospital of Pediatrics and Adolescent Medicine, Ruhr-University Bochum, Bochum, Germany; 3 Pediatric Clinic Dortmund, Dortmund, Germany; 4 Laboratory for Translational Hormone Analytics, Peptide Hormone & Immunoassay Unit, Pediatric Endocrinology & Diabetology, Center of Child and Adolescent Medicine, Justus Liebig University, Giessen, Germany

**Keywords:** Acid-base status, albumin-creatinine ratio, albuminuria, ammonium excretion, estimated glomerular filtration rate, net acid excretion, net endogenous acid production, urinary pH

## Abstract

**Rationale & Objective:**

Metabolic acidosis and reduced serum bicarbonate levels are known to contribute to kidney disease progression. Whether habitual high net endogenous acid production early in life may have consequences for long-term kidney function is not known. To date, no longitudinal study has examined the relationships between regular net acid excretion (NAE) during childhood and adolescence and later adult albumin excretion, estimated glomerular filtration rate (eGFR), and creatinine clearance (CL_Cr_).

**Study Design:**

Open cohort study examining healthy children from infancy to adulthood.

**Setting & Participants:**

Study participants with multiple 24-hour urine collections between ages 3 and 17 years who provided a blood sample between ages 18 and 35 years.

**Exposure:**

Pre-adulthood NAE and its components ammonium excretion, titratable acidity, and pH.

**Outcomes:**

Adults’ eGFR, CL_Cr_, and albumin-creatinine ratio (ACR).

**Analytical Approach:**

Sex- and age-stratified standard deviation scores calculated for anthropometric and urinary biomarkers were averaged for each individual, and the relationships between urinary exposures and adulthood eGFR, CL_Cr_, and ACR were examined using multiple linear regression analysis. Regression models were adjusted for pre-adulthood nutrition-related renal biomarker standard deviation scores and various adult parameters.

**Results:**

In fully adjusted models, children’s and adolescents’ repeatedly measured renal NAE and titratable acidity had a strong inverse association and their 24-hour urinary pH had a highly significant positive association with eGFR in adulthood. Similar findings were seen for CL_Cr_. Pre-adulthood ammonium excretion had a significant positive relationship only with ACR in adulthood.

**Limitations:**

Outcomes determined only once.

**Conclusions:**

These exclusively biomarker-based findings strongly suggest that habitually increased NAE and corresponding low urinary pH values in the normal range in childhood and adolescence are already related to incipient signs of kidney function decline in adulthood. Hence, as an important kidney health prevention measure, habitual consumption of alkalizing fruit- and vegetable-rich diets with clear ammoniagenic and NAE-lowering efficiency should start early in childhood.

## Plain-Language Summary

Metabolic acidosis negatively affects kidney function. Whether habitually high renal net acid excretion early in life may already negatively affect kidney function in the long run is not known. By examining healthy children’s and adolescents’ acid excretion and urinary pH values in 24-hour urine samples, repeatedly collected during ages 3-17 years, we demonstrated that higher acid excretion and lower urinary pH are adversely related to glomerular filtration rate, creatinine clearance, and partially to albuminuria later in adulthood. These findings strongly suggest that regularly increased acid loads to the body during childhood and adolescence are already involved in adversely influencing kidney function, indicating the importance of early alkalizing fruit- and vegetable-rich nutrition for long-term kidney health.

Urinary albumin excretion and glomerular filtration rate (GFR) are major indicators to assess kidney function, and hence, they are the most frequently used measures for detection and staging of acute and chronic kidney disease (CKD).^1^ Common and cost-effective laboratory tests routinely conducted for kidney function assessment in clinical settings are measurements of serum creatinine for the determination of estimated glomerular filtration rate (eGFR) and of urinary albumin for the detection of albuminuria. Albuminuria and lowered GFR during CKD^2^ accompany an increased prevalence of metabolic acidosis.^3^^,^^4^ Meanwhile, evidence that metabolic acidosis itself is a contributor to kidney disease progression is increasing.^5^^,^^6^

Suspected mechanisms that contribute to acid-mediated kidney injury are actually compensatory responses that are invoked to prevent or attenuate the severity of acidosis.^6^^,^^7^ These mechanisms include an increased secretion of endothelin-1,^8^ increased activities of the renin-angiotensin-aldosterone system, and enhanced renal ammonium (NH_4_) production,^5^ all intended to facilitate acid excretion by the kidneys.^7^ Enhanced renal ammonium production is also the major physiological kidney response to a high dietary acid load. Surplus acid equivalents (H^+^) from diets with a high potential renal acid load are renally buffered by either phosphate or ammonia and excreted as titratable acidity (TA) or ammonium. Elevated renal tissue ammonium levels, which commonly result from a reduced number of nephrons in CKD, induce activation of the alternative complement system and complement-driven inflammation, which may further accelerate the decline in kidney function in patients with CKD.^9^ In people with healthy kidneys, elevations of ammonia levels per nephron can only occur in case of excessive net endogenous acid production, for example, due to severe losses of alkali (bicarbonate) or high dietary acid intake. Human observational and animal studies suggest that lowered serum bicarbonate levels as well as diets with high potential renal acid load, which are known to raise renal ammonium concentrations and net acid excretion (NAE), may contribute to more rapid kidney function decline in the long run, even under mostly healthy or CKD-free conditions.^9^, ^10^, ^11^, ^12^, ^13^

Recently, observational evidence has revealed enhancement of renal inflammatory processes in adults in whom NAE and ammonium excretion was repeatedly high during childhood and adolescence, but who were otherwise healthy.^14^ However, data are lacking on whether habitual, high endogenous acid production during childhood and adolescence, albeit in the normal range, affects kidney function in the long run. We therefore prospectively examined potential consequences of repeated measurements of higher NAE and its components ammonium, TA, and 24-hour urinary pH during growth on eGFR, creatinine clearance (CL_Cr_), and albuminuria in adulthood.

## Methods

### Study Population

Healthy individuals participating in the Dortmund Nutritional and Anthropometric Longitudinally Designed (DONALD) study were examined. Initiated in 1985, this open cohort study collects data on growth, dietary intakes, and urinary metabolite excretions of healthy volunteers from infancy through childhood and adolescence to adulthood.^15^ From the age of 3-4 years onward, participating children provide a 24-hour urine sample once a year.^16^ At age 18 years, and then at multiyear intervals, blood samples are also collected. All assessments and examinations are conducted with written consent from both parents of participating children and from the children (as adults), and the study protocol was approved by the ethics committee of the University of Bonn, Germany (approval Nos. 098/06 and 185/20). All examinations were performed in accordance with the guidelines of the Declaration of Helsinki.

A total of 572 individuals were initially identified, each with at least 5 eligible 24-hour urine collections available before adulthood—specifically, a minimum of 2 samples collected during childhood (ages 3-8 years) and at least 3 from the ages of 9 to 17 years. Among these 572 subjects, participants were selected for whom a minimum of 3 renal NAE measurements from 24-hour urine collections had been performed (n = 523). For analyzing the relationship between average pre-adulthood NAE level and adult eGFR, a total of 354 of the latter 523 participants were finally included, all with an available blood collection between ages 18 and 35 years, and measurements of kidney health-related parameters, including creatinine for eGFR calculation, were performed. The average number of pre-adulthood 24-hour NAE measurements of these 354 participants was almost 10 per person. Of these 354 participants, 24-hour urine samples were collected in adulthood from 216 participants. Moreover, of all 354 participants, only 152 could be further included for examining the outcome of albuminuria. Separate spot urine samples had been collected from these 152 participants between ages 18 and 35 years, which were frozen immediately at −80°C for subsequent albumin and creatinine measurements.

### Anthropometric Measurements

Anthropometric data were measured by trained nurses following standardized protocols. Body weight was recorded to the nearest 0.1 kg using a digital scale (Seca 753E; Seca Weighing and Measuring Systems), and standing height was measured to the nearest 0.1 cm using a stadiometer (Harpenden; Holtain Ltd). Body mass index, body surface area (BSA)^17^, and adult fat free mass^18^ were calculated according to established formulas as outlined in Item S1.

### Blood Measurements

Venous blood samples (<20 mL) were collected after overnight fasting, centrifuged at 4°C for 15 minutes, and then stored at −80°C. Serum levels of uric acid, urea, and high-density and low-density lipoprotein cholesterol, as well as creatinine, were measured using a Roche/Hitachi Cobas c311 analyzer (Roche Diagnostics) at the clinical laboratory of the pediatric clinic Dortmund, Germany. Plasma insulin concentrations were analyzed using an immunoradiometric assay (DRG Diagnostics) at the laboratory for translational hormone analytics of the University of Giessen. eGFR was calculated based on the creatinine-based equations published by the Chronic Kidney Disease Epidemiology Collaboration,^19^ and CL_Cr_ was determined according to the formula provided in Item S1.

### Urinary Measurements

Children participating in the DONALD study and their parents were provided with detailed instructions on how to collect a 24-hour urine sample at home, using preservative-free, Extran-cleaned (Extran, MA03; Merck) 1-L plastic containers. The urine samples were stored at −18 to −20°C until analysis.^20^ Creatinine excretion both in 24-hour and spot samples were measured using a creatinine analyzer (Beckman-2; Beckman Instruments) using the kinetic Jaffé method. To minimize errors related to 24-hour urine collection, samples with creatinine excretion rates <0.1 mmol/kg/d were not included in the analyses.^21^ Urinary urea was analyzed by the Urease-Berthelot method (Randox Laboratories). All acid-base analytes, including ammonium (mmol/L), bicarbonate (mmol/L), TA (mEq/L), and 24-hour urinary pH, were determined using the 3-phase acid-base titration method^22^ with a Mettler Toledo endpoint titrator. NAE was calculated by summing titratable acid and ammonium, then subtracting bicarbonate. Calcium, sodium, and potassium excretion in urine were determined by flame atomic absorption spectrometry (Perkin Elmer 1100 Spectrometer). Albumin concentration in spot samples were measured using a Roche/Hitachi Cobas c311 analyzer at the clinic laboratory of the pediatric clinic Dortmund, Germany.

### Statistical Analysis

All statistical analyses were performed using SAS software (SAS Institute Inc; version 9.4), and *P* values < 0.05 were considered statistically significant. The Shapiro-Wilk test and Q-Q plots were used to evaluate the normality of the variables. To describe the general characteristics of the study participants, normally distributed variables are presented as mean (± standard deviation [SD]), while nonnormally distributed variables are shown as median (25th, 75th percentiles). To compare the first and last assessments, for normally distributed variables, paired *t* test was used, whereas the Wilcoxon signed-rank test was applied for nonnormally distributed variables.

Internal standardized scores (mean = 0, SD = 1) stratified by sex and age were calculated for all anthropometric and 24-hour urinary biomarkers. The respective standard deviation scores (SDSs) were averaged for each individual and included in the analyses as the individual’s arithmetic mean of SDS. Multiple linear regression models (PROC GLM) were employed to investigate the prospective associations of the exposure NAE and its major determinants ammonium and TA and the 24-hour urinary pH during childhood and adolescence, with the outcomes eGFR, CL_Cr_, and albuminuria in adulthood.

Because no sex-specific interactions were found (*P* > 0.1), all regression analyses were performed without sex stratification. The 4 key assumptions of multiple linear regression analysis, which are normal distribution of residuals, non-multicollinearity, linearity, and homoscedasticity, were checked and none of them were violated. Covariates were included and remained in the models if they met any of the following criteria: (1) they altered the β coefficient of the exposure variable more than 10%, indicating that they modified the association between the exposition variables (NAE-SDS, NH_4_-SDS, TA-SDS, and pH-SDS) and the outcomes eGFR, CL_Cr_, or the albumin-creatinine ratio (ACR); (2) they enhanced the overall explained variability of the model; or (3) they showed a significant independent association with eGFR, CL_Cr_, or ACR (*P* < 0.05).

**Figure 1:**
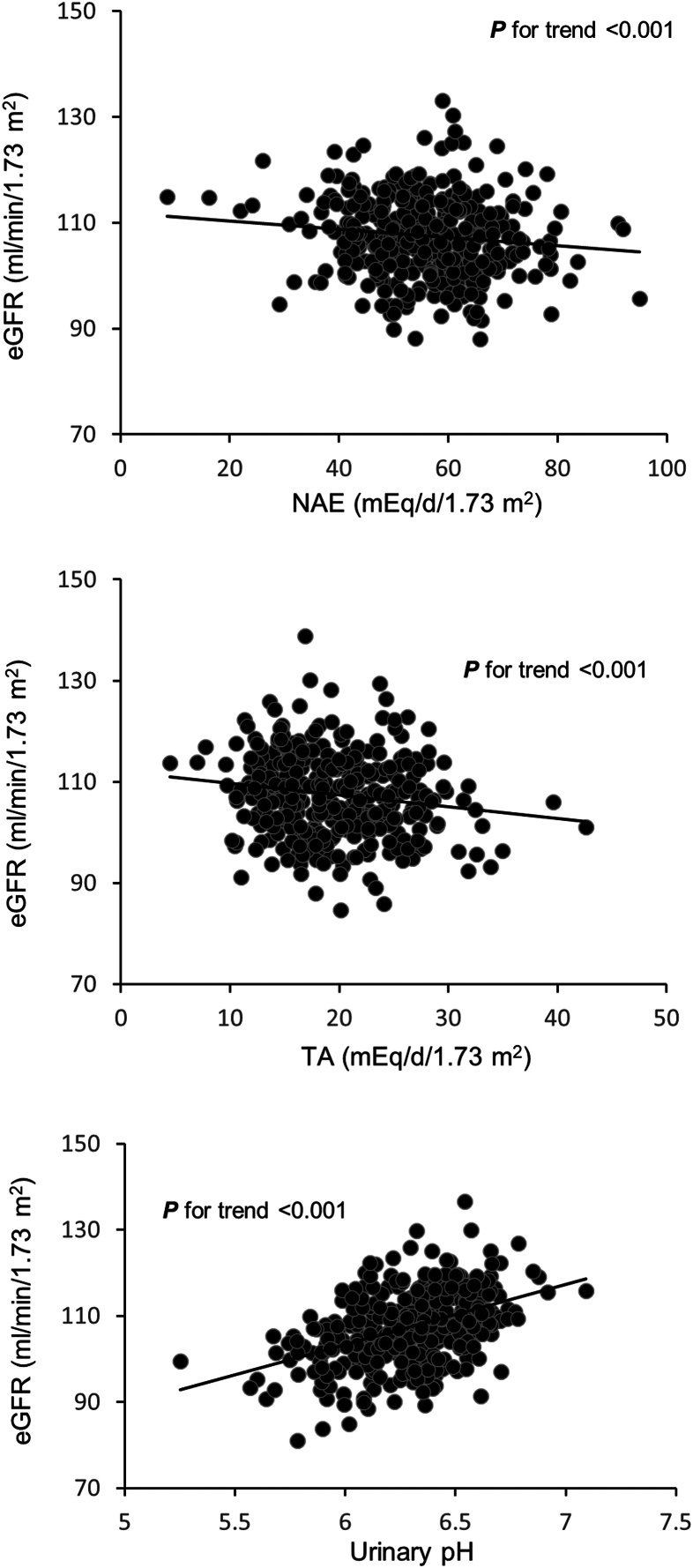
Prospective relationships of healthy individuals’ means of 24-hour NAE and 24-hour titratable acidity (both corrected for body surface area) as well as of 24-hour urinary pH during growth with eGFR in adulthood (n = 354). Predicted values of eGFR were derived from multiple linear regressions after adjustment for sex, 24-hour urinary excretions of urea nitrogen, creatinine, and potassium during growth, and fat free mass in adulthood. eGFR, estimated glomerular filtration rate; NAE, net acid excretion; TA, titratable acid excretion.

The PROC GLM models of the outcomes eGFR, CL_Cr_, and ACR for the predictors NAE-SDS, NH_4_-SDS, TA-SDS, and pH-SDS were adjusted in 3 steps for the following covariates: sex, adult age, pre-adulthood urinary urea nitrogen, urinary creatinine, urinary volume, urinary calcium, and urinary potassium, as well as the adult blood parameters insulin, urea, uric acid, and high-density lipoprotein cholesterol. In the final models, only covariates having a *P* value < 0.2 were eventually included. To further ensure childhood and adolescent net endogenous acid production was a potential influencing factor of adult eGFR, regression models were re-evaluated with pre-adulthood individual means of BSA-corrected NAE, BSA-corrected TA, and 24-hour urinary pH as exposure variables (Fig 1). A sensitivity analysis was additionally performed in the reduced sample of 216 participants for whom a 24-hour urine sample was also available in adulthood. This allowed to control for adult urinary urea nitrogen excretion, the renal biomarker of protein intake, because high protein intake can temporally increase eGFR.

## Results

Long-term general characteristics of the study participants during growth, that is, anthropometric and 24-hour urinary parameters as well as their adult characteristics, are shown in Table 1. Mean age of the 354 study participants at the first assessment was 4 years and 16 years at the last. All anthropometric data and 24-hour urinary excretion rates except BSA-corrected NAE differed significantly between first and last assessment (Table 1). At the last compared to the first assessment, urinary pH had decreased by 0.1, whereas absolute excretion rates of NAE, ammonium, and TA had increased substantially, for example, by 28.1 mEq/d on average for NAE (Table 1). Out of the 354 participants, only 3 had on 1 occasion—and 1 participant on 2 occasions—an NAE exceeding 135 mEq/d/1.73 m^2^ (Fig S1). This NAE level is indicative of an exaggerated acid load, which corresponds to a low 24-hour urinary pH of around 5.5.^23^ Obviously, none of the participants had manifested any chronic high acid load-induced eubicarbonatemic or overt metabolic acidosis during childhood and adolescence.

**Table 1:** Characteristics of Participants During Growth and Adulthood

	Longitudinal Overview^a^	
	First Assessment	Last Assessment^b^
Childhood and adolescence		
n (male/female)	354 (177/177)	354 (177/177)
Age, y	4.0 (3.4, 5.0)	16.1 (13.9, 17.0)
Weight, kg	18.1 ± 3.5	60.5 ± 16.0
Height, cm	107.1 ± 8.5	168.9 ± 13.8
BMI, kg/m^2^	15.6 ± 1.2	20.8 ± 3.5
BSA, m^2^	0.73 ± 0.1	1.69 ± 0.3
NAE, mEq/d	23.7 ± 12.2	51.8 ± 25.5
NAE-BSA, mEq/d/1.73 m^2^	56.1 ± 26.8	52.8 ± 22.9
NH_4_, mmol/d	19.3 ± 5.9	40.6 ± 14.1
NH_4_-BSA, mmol/d/1.73 m^2^	45.8 ±12.1	41.6 ± 12.0
TA, mEq/d	8.5 ± 5.0	18.1 ± 10.0
TA-BSA, mEq/d/1.73 m^2^	20.0 ± 11.1	18.4 ± 9.0
Urinary pH	6.4 ± 0.5	6.3 ± 0.5
Urea-N, mmol/d	169.7 ± 52.7	340.5 ± 114.2
Volume, mL/d	507.4 (370.0, 666.3)	1,192.8 (868.1, 1664.3)
Creatinine, mmol/d	2.7 ± 0.7	10.9 ± 3.7
Na, mmol/d	58.8 ± 23.9	129.1 ± 58.0
K, mmol/d	33.5 ± 11.0	54.1 ± 20.5
Ca, mmol/d	0.8 (0.5, 1.3)	2.6 (1.5, 3.9)
Adulthood		
Age, y	20.8 (18.1, 23.5)	—
Weight, kg	73.0 ± 16.0	—
Height, cm	176.8 ± 9.4	—
BMI, kg/m^2^	23.2 ± 4.1	—
BSA, m^2^	1.89 ± 0.2	—
FFM, kg	52.4 ± 12.3	—
eGFR, mL/min/1.73 m^2^	107.6 ± 19.1	—
CL_Cr_, mL/min^c^	119.8 ± 36.0	—
Albuminuria, mg/g^d^	2.9 (1.4, 5.4)	—
Insulin, μIU/mL	11.2 (8.4, 14.2)	—
Uric acid, mg/dL	5.2 ± 1.2	—
Urea, mg/dL	26.8 ± 7.7	—
HDL, mg/dL	57.8 ± 15.1	—
LDL, mg/dL	96.1 ± 29.7	—
HDL/LDL	0.7 ± 0.3	—

*Note*: Values shown are means ± SDs if normally distributed or medians (25th, 75th percentiles) if not normally distributed.

Abbreviations: BMI, body mass index; BSA, body surface area; Ca, 24-hour calcium excretion; CL_Cr_, creatinine clearance; eGFR, estimated glomerular filtration rate; FFM, fat free mass; HDL, high-density lipoprotein cholesterol; K, 24-hour potassium excretion; LDL; low- density lipoprotein cholesterol; Na, 24-hour sodium excretion; NAE, 24-hour net acid excretion; NH_4_, 24-hour ammonium excretion; SD, standard deviation; TA; 24-hour titratable acid excretion; Urea-N, 24-hour urea nitrogen excretion.

a The first and the last urine sample of each participant who had at least 2 eligible urine samples collected between age 3-8 years, and at least 1 eligible urine samples collected between age 9-17 years (n = 354; average number of urine samples collected per person = 9.7).

b Differences between the first and last assessments were tested with paired *t* test for normally distributed variables and Wilcoxon signed-rank test for nonnormally distributed ones (all differences *P* < 0.001, except for 24-hour urinary pH [*P* = 0.005], BSA-corrected 24-hour urinary NAE [*P* = 0.08], and BSA-corrected 24-hour urinary TA [*P* = 0.02]).

c Assessment of creatinine clearance was performed in 216 participants.

d Measurements of albuminuria (albumin concentration divided by creatinine concentration) were available for 152 participants.

Results of multiple linear regression analyses for the associations of 24-hour biomarker-based assessed urinary NAE, ammonium, TA, and pH during childhood and adolescence with eGFR, CL_Cr_, and ACR in adulthood are summarized in Table 2, Table 3, Table 4, respectively.

**Table 2:** Prospective Relationships of Renal Net Endogenous Acid Production (NAE) and its 24-hour Urinary Components (Ammonium, Titratable Acidity, and pH) During Growth with eGFR in Adulthood (Analyses Performed in 354 Healthy Individuals)

	β^a^ (95% CI)	*R**^2^*	*P*
NAE-SDS			
Model I	−1.36 (−5.02, 2.29)	0.06	0.46
Model II	−7.49 (−12.73, −2.25)	0.15	0.005
Model III	−9.95 (−14.68, −5.21)	0.27	<0.001
NH_4_-SDS			
Model I	3.55 (0.21, 6.89)	0.07	0.04
Model II	2.34 (−2.50, 7.17)	0.14	0.34
Model III	−1.13 (−5.82, 3.56)	0.25	0.63
TA-SDS			
Model I	−0.88 (−1.33, −0.43)	0.09	<0.001
Model II	−1.63 (−2.14, −1.12)	0.21	<0.001
Model III	−1.51 (−2.00, −1.02)	0.31	<0.001
pH-SDS			
Model I	6.14 (2.60, 9.68)	0.09	<0.001
Model II	10.25 (6.52, 13.97)	0.18	<0.001
Model III	9.51 (6.00, 13.01)	0.29	<0.001

*Note*: Results obtained from stepwise multilinear regression analyses. Model I adjusted for sex and adult age. Model II adjusted for variables in model I plus childhood and adolescent means of SDSs of nutrition-related 24-hour urinary biomarkers, potentially interacting with acid-base status (urea nitrogen, creatinine, and potassium). For predictors NAE-SDS and NH_4_-SDS, 24-hour urinary volume (also fulfilling the inclusion criteria), and for TA-SDS, 24-hour urinary calcium excretion were additionally included. Model III adjusted for all variables in model II plus adults’ blood-derived parameters (insulin, uric acid, urea, and HDL) and adults’ FFM to control for muscle mass and body composition. After removing all covariates with *P* values exceeding 0.2, the respective final models III were adjusted for the following variables: sex, 24-hour urinary nitrogen urea, 24-hour urinary potassium, 24-hour urinary creatinine, blood urea, blood uric acid, insulin, HDL, and adult FFM; the NH_4_-SDS predictor model III was additionally adjusted for urine volume and the TA-SDS model III for urinary calcium. Controlling for the indicator of adult muscularity FFM did improve the models’ *R*^2^ and was itself an independent (*P* < 0.05) predictor of eGFR in the NAE-SDS, TA-SDS, and pH predictor models III.

Abbreviations: BSA, body surface area; CI, confidence interval; eGFR, estimated glomerular filtration rate; FFM, fat free mass; HDL, high-density lipoprotein cholesterol; NAE-SDS, NH_4_-SDS, TA-SDS: individual means of standard deviation scores of children’s and adolescents’ 24-hour net acid excretion, 24-hour ammonium excretion, 24-hour titratable acid excretion; SD, standard deviation; SDS, standard deviation score.

a β values refer to the change of eGFR (mL/min/1.73 m^2^) per one SD change of the respective exposure variable NAE-SDS, NH4-SDS, TA-SDS, and 24-hour urinary pH. One SD of the exposure variables approximates to a mean change during pre-adulthood of 12.6 mEq/d/1.73 m^2^ for NAE, 7.1 mmol/d/1.73 m^2^ for NH_4_, 5.5 mEq/d/1.73 m^2^ for TA, and 0.26 pH units for 24-hour urinary pH. Data represent the SDs of the overall means of participants’ individual pre-adulthood mean values of the respective BSA-corrected exposures and of urinary pH. Accordingly, a pre-adulthood urinary pH that is habitually ∼0.26 pH units higher than the average 24-hour urinary pH of 6.3 (Table 1) would accompany an increase in adulthood eGFR of ∼9.51 mL/min/1.73 m^2^.

**Table 3:** Prospective Relationships of Renal Net Endogenous Acid Production (NAE) and its 24-hour Urinary Components (Ammonium, Titratable Acidity, and pH) During Growth with Creatinine Clearance in Adulthood (Analyses Performed in 216 Healthy Individuals)

	β^a^ (95% CI)	*R*^*2*^	*P*
NAE-SDS			
Model I	7.03 (−1.68, 15.75)	0.14	0.11
Model II	−14.40 (−26.25, −2.55)	0.25	0.02
Model III	−11.74 (−22.17, −1.31)	0.34	0.03
NH_4_-SDS			
Model I	15.14 (7.69, 22.59)	0.20	<0.001
Model II	5.91 (−5.26, 17.08)	0.23	0.30
Model III	5.80 (−1.53, 13.13)	0.33	0.12
TA-SDS			
Model I	−0.18 (−1.24, 0.87)	0.13	0.73
Model II	−2.36 (−3.54, −1.18)	0.28	0.001
Model III	−2.27 (−3.37, −1.17)	0.37	<0.001
pH-SDS			
Model I	8.02 (−0,54, 16.58)	0.15	0.07
Model II	14.17 (5.63, 22.71)	0.27	0.001
Model III	11.60 (3.88, 19.32)	0.35	0.003

Note: Results obtained from stepwise multilinear regression analyses. Model I adjusted for sex and adult age. Model II adjusted for variables in model I plus childhood and adolescent means of SDSs of nutrition-related 24-hour urinary biomarkers, potentially interacting with acid-base status (urea nitrogen, creatinine, potassium and calcium). Model III adjusted for all variables in model II plus adults’ blood-derived parameters (insulin, uric acid, urea, and HDL) and adults’ FFM to control for muscle mass and body composition. After removing all covariates with *P* values exceeding 0.2, the respective final models III were adjusted for the following variables: 24-hour urinary calcium, blood uric acid, HDL, and adult FFM; the NH_4_-SDS predictor model III was additionally adjusted for blood urea and the TA-SDS, NAE-SDS, and pH-SDS models III for urinary urea nitrogen. Controlling for the indicator of adult muscularity FFM did improve models’ *R*^2^ and was itself an independent (*P* < 0.001) predictor of creatinine clearance in all 4 models III.

Abbreviations: BSA, body surface area; CI, confidence interval; eGFR, estimated glomerular filtration rate; FFM, fat free mass; HDL, high-density lipoprotein cholesterol; NAE-SDS, NH_4_-SDS, TA-SDS: individual means of standard deviation scores of children’s and adolescents’ 24-hour net acid excretion, 24-h ammonium excretion, 24-h titratable acid excretion; SD, standard deviation; SDS, standard deviation score.

a β-values refer to the change of creatinine clearance (mL/min) per 1 SD change of the respective exposure variable NAE-SDS, NH4-SDS, TA-SDS, and 24-hour urinary pH. One SD of the exposure variables approximates to a mean change during pre-adulthood of 12.6 mEq/d/1.73 m^2^ for NAE, 7.1 mmol/d/1.73 m^2^ for NH_4_, 5.5 mEq/d/1.73 m^2^ for TA, and 0.26 pH units for 24-hour urinary pH. Data represent the SDs of the overall means of participants’ individual pre-adulthood mean values of the respective BSA-corrected exposures and of urinary pH. Accordingly, a pre-adulthood urinary pH that is habitually ∼0.26 pH units higher than the average 24-hour urinary pH of 6.3 (Table 1) would accompany an increase in adulthood creatinine clearance of ∼11.6 mL/min.

**Table 4:** Prospective Relationships of Renal Net Endogenous Acid Production (NAE) and its 24-hour Urinary Components (Ammonium, Titratable Acidity, and pH) During Growth with Albuminuria in Adulthood (Analyses Performed in 152 Healthy Individuals)

	β^a^ (95% CI)	*R*^*2*^	*P*
NAE-SDS			
Model I	−1.10 (−1.51, 1.26)	0.02	0.55
Model II	1.20 (−1.32, 1.91)	0.08	0.43
Model III	1.10 (−1.29, 1.55)	0.11	0.60
NH_4_-SDS			
Model I	−1.00 (−1.35, 1.32)	0.02	0.97
Model II	1.66 (1.10, 2.51)	0.11	0.01
Model III	1.70 (1.12, 2.57)	0.14	0.01
TA-SDS			
Model I	−1.17 (−1.62, 1.15)	0.03	0.28
Model II	−1.05 (−1.66, 1.48)	0.08	0.82
Model III	−1.02 (−1.45, 1.41)	0.10	0.92
pH-SDS			
Model I	1.02 (−1.35, 1.38)	0.02	0.92
Model II	1.05 (−1.35, 1.48)	0.08	0.79
Model III	1.05 (−1.35, 1.48)	0.10	0.81

*Note*: Results were obtained from stepwise multilinear regression analyses using log_10_ transformed albumin-creatinine ratios. The β coefficients shown have been de-logarithmized, accounting for the fact that the 95% CIs (that have also been de-logarithmized) are asymmetric. Model I adjusted for sex and adult age. Model II adjusted for variables in model I plus childhood and adolescent means of SDSs of nutrition-related 24-hour urinary biomarkers, potentially interacting with acid-base status (urea nitrogen, creatinine, and volume). For the predictor pH, 24-hour urinary urea nitrogen did not fulfill the inclusion criteria and was not included. Model III adjusted for all variables in model II plus adults’ blood-derived parameters (eGFR and HDL) and adults’ FFM to control for muscle mass and body composition. For the predictor pH, insulin also fulfilled the inclusion criteria and was initially included in model III. After removing all covariates with *P* values exceeding 0.2, the respective final models III were adjusted for the following variables: sex, pre-adulthood 24-hour urinary creatinine and urinary volume as well as eGFR and HDL; the NH_4_-SDS predictor model III was additionally adjusted for 24-hour urinary urea nitrogen, and the NAE-SDS predictor model III also included adult FFM.

Abbreviations: BSA, body surface area; CI, confidence interval; eGFR, estimated glomerular filtration rate; FFM, fat free mass; HDL, high-density lipoprotein cholesterol; NAE-SDS, NH_4_-SDS, TA-SDS: individual means of standard deviation scores of children’s and adolescents’ 24-h net acid excretion, 24-h ammonium excretion, 24-h titratable acid excretion; SD, standard deviation; SDS, standard deviation score.

a β-values refer to the change of albuminuria (mg/g) per 1 SD change of the respective exposure variables NAE-SDS, NH4-SDS, TA-SDS, and 24-hour urinary pH. One SD of the exposure variables approximates to a mean change during pre-adulthood of 12.6 mEq/d/1.73 m^2^ for NAE, 7.1 mmol/d/1.73 m^2^ for NH_4_, 5.5 mEq/d/1.73 m^2^ for TA, and 0.26 pH units for 24-hour urinary pH. Data represent the SDs of the overall means of participants’ individual pre-adulthood mean values of the respective BSA-corrected exposures and of urinary pH. Accordingly, a pre-adulthood ammonium excretion that is habitually ∼5.5 mEq/d/1.73 m^2^ higher than the average ammonium excretion of ∼41-46 mEq/d/1.73 m^2^ (Table 1) would accompany an increase in adulthood albuminuria of ∼1.7 mg/g.

After adjusting for growth-related nutritional biomarkers and adulthood covariates including fat free mass, highly significant inverse associations were observed between long-term pre-adulthood NAE and TA and later adulthood eGFR (Table 2). Corresponding results were seen for CL_Cr_ in the subsample of participants with an additional adult 24-hour urine collection (Table 3). In line with this, strong positive associations were found between children’s and adolescents’ urinary pH and their eGFR and CL_Cr_ in adulthood (Tables 2 and 3), whereas no significant relationship was seen between ammonium and eGFR or CL_Cr_. Of note, comparable results were obtained for eGFR after running the regression analyses again, but now with the individual means of directly measured 24-hour urinary pH and BSA-corrected NAE (mEq/d/1.73 m^2^) and TA (mEq/d/1.73 m^2^) (Fig 1), instead of using exposures’ SDS. Ammonium (mmol/d/1.73 m^2^) was again not significant (data not shown). Ammonium excretion was the only acid-base component that significantly associated with adults’ albuminuria after allowing for both specific pre-adulthood and adulthood covariates (*P* = 0.01, Table 4).

The sensitivity analysis (Fig S2), which additionally allowed for adults’ 24-hour urinary urea nitrogen excretion (the marker of protein intake), confirmed the relationships between adults’ eGFR and pre-adulthood renal NAE, TA, and urinary pH.

## Discussion

Using biomarker-based, prospective examinations of healthy individuals from childhood to young adulthood, the present longitudinal study provides evidence that habitual high acid loads to the body during growth may relevantly contribute to the initiation of kidney function decline in the long term.

Repeatedly measured 24-hour urinary ammonium excretion rates of children and adolescents, consuming normal diets, were significantly associated with the urinary ACR determined later in their adulthood. Their habitual renal NAE and urinary TA was associated significantly inversely with their adult eGFR and CL_Cr_. In addition, the children’s and adolescents’ 24-hour urinary pH was highly significantly related with both eGFR and CL_Cr_ in adulthood.

Despite clear associations of eGFR and CL_Cr_ with NAE, TA, and pH, no significant relationship was discernible between eGFR and ammonium excretion (the major component of net endogenous acid production). This finding may be most plausibly explained by the fact that along with children’s higher acid equivalent-yielding protein intake, renal acid excretion capacity, that is, the renal ammonium production potential,^24^ acutely increases. What probably also increases is the overall GFR capacity during growth due to the distinct anabolic IGF-1 effects triggered by habitual high protein intakes.^25^, ^26^, ^27^ IGF-1 is one of the major anabolic stimulators of kidney size and kidney function.^28^ Thus, a potentially increased kidney size in “habitual high-protein consumers” would constitute an unmeasured confounder not allowing to clearly unmask the potential relevance of pre-adulthood ammonium production on the adult kidney function marker GFR.

In CKD patients, reduced GFR and metabolic acidosis commonly co-occur with increases in albumin excretion. An only modestly elevated albumin excretion rate—even within the normoalbuminuric range—has been reported to identify risks for incident CKD, kidney failure, cardiovascular disease, and all-cause mortality not only in patients with CKD but also in persons with relatively preserved kidney function.^29^^,^^30^

One major cause of the development of albuminuria is an increase in the permeability of the glomerular filtration barrier, that is, a function loss of the 3-layered renal filtration barrier eventually resulting in loss of albumin into urine across the wall of small renal capillaries.^31^ The exact operating principles of the complex renal filter and the reason for its altered permeability in kidney diseases have not been fully clarified.^31^ A second important cause for increased losses of albumin in urine is defective reabsorption of albumin from the glomerular ultrafiltrate by the proximal tubular cells.^32^, ^33^, ^34^ Studies in humans with defects in tubular reabsorption of albumin have shown that this failure goes along with a urinary loss of albumin up to approximately 1,000 mg/d,^34^ indicating that in the healthy state, a considerable amount of albumin physiologically leaks through the glomerulus. Functional metabolic derangements of the nephrons’ proximal tubular segment, which go hand in hand with increased albumin excretion rates, can be driven by various causes—for example, in patients with diabetes mellitus or obesity—by glucose overload, increased production of reactive oxygen species, and/or proinflammatory processes.^35^^,^^36^

An increase in inflammatory processes might also be the underlying mechanism for the observed significant relationship between consistently high ammonium excretion rates in our young study participants and the higher ACRs later in their adulthood.

Different research groups have provided experimental evidence in tissue, animal, and human studies that elevated intrarenal concentrations of the basically tissue-toxic ammonia can induce tubulointerstitial inflammation processes, mediated via activation of the alternative complement pathway.^13^^,^^37^^,^^38^

Augmented intrarenal levels of ammonia may activate the proximal tubular cells’ complement cascade, leading to tubular cell activation, cell injury, and the release of proinflammatory cytokines,^39^ which eventually mediate and contribute to progressive tubulointerstitial damage.^39^^,^^40^ This would compromise tubular albumin reabsorption, particularly in view of the fact that renal ammonium production is principally taking place in proximal tubular cells.

In accordance with probable proximal tubular tissue damage caused by long-term high ammonia production, potentially mediated in the last step via inflammatory processes, a recent study by our group showed that habitual high ammonium excretion in healthy children associates with these children’s circulating renal tubular injury marker interleukin-18 later in adulthood.^14^

However, what must be kept in mind is that renal ammoniagenesis, in principle, is an essential physiological process ensuring the buffering of excess protons that need to be renally excreted, thereby preserving basal bicarbonate regeneration. Accordingly, regardless of the origin, reduced ammonia production capability, which results in an inadequate urinary ammonium concentration for a given acid load, will probably lead to metabolic acidosis. In patients with CKD, metabolic acidosis primarily results from insufficient ammonium excretion.^41^ Therefore, in these patients with metabolic acidosis, no positive association between renal ammonium excretion and albuminuria can be expected. Maintenance of adequate renal ammonia metabolism appears on the one hand to be protective for patients with CKD, whereas on the other hand, ammonia itself may directly contribute to CKD development or CKD progression by activating complement.^41^

A number of clinical trials conducted in patients with CKD have examined the impact of correcting metabolic acidosis with alkalizing measures on CKD progression. A systematic review and meta-analysis of these studies provided low-to-moderate certainty evidence indicating that oral alkali supplementation or a reduction in dietary acid intake can slow the rate of kidney function decline and potentially reduce the risk of end-stage kidney disease in these patients.^42^ However, some controversy still exists on whether sodium bicarbonate administration or nutritional therapy with fruits and vegetables is safer and what might be the potential negative effects of alkalization.^43^

In addition, in large cross-sectional studies, for example, NHANES,^10^ Jackson Heart,^11^ and a Japanese Cohort study,^12^ which examined the general population and not specifically patients with CKD, significant positive relationships of albuminuria with estimates of net endogenous acid production and inverse relationships of the latter with eGFR have been reported. In these studies, the exposures, that is, daily net endogenous acid production rates, were estimated from self-reported dietary intakes, whereas in our study the laboratory gold standard measurements NAE and ammonium excretion were applied. Thus, our long-term biomarker analyses over many years pre-adulthood substantiate a potential incipient impairment of the major kidney function parameters albumin excretion and eGFR in young adulthood through acid loads regularly requiring higher ammonium production rates during childhood and adolescence.

Strengths of the present study—apart from its prospective longitudinal design—are particularly the examination of 24-hour urine samples repeatedly collected from healthy young persons over an average observation period of 10 years before adulthood and the use of the gold standard measurement NAE to provide a biomarker-based characterization of acid loads on the body. Further strengths are that we adjusted for adult fat free mass (an index of muscularity), as muscle mass interferes with the creatinine-based eGFR assessment. In addition, we allowed for biomarker-based assessments of protein intake, which is a potential confounder of eGFR as well.

Limitations of the study were the measurement of the ACR in spot samples instead of analyzing albumin excretion in 24-hour samples and the facts that outcomes in adulthood were determined only once and no cystatin C measurements were available to additionally calculate a cystatin C-based eGFR. For an extra assessment of a possible progression of kidney function decline through the calculation of eGFR slope, we could unfortunately not yet make use of enough second-time collected adult blood samples. In addition, no childhood baseline data were available for the outcomes due to the exclusively noninvasive nature of the DONALD study throughout growth. Also, the number of examined individuals was rather limited owing to the fact that attendance of participants in longitudinal studies lasting >20 years is intrinsically low. In addition, the present findings were obtained in a cohort of children of European descent and are not necessarily applicable to other ethnicities.

In summary, this study prospectively examined biomarkers over a long-term observation period to ascertain the relationship of habitual high net endogenous acid production during growth with the 3 kidney function markers, eGFR, CL_Cr_, and albumin excretion, assessed in young adulthood. Children’s and youth’s NAE, TA, and urinary pH were all strongly related (NAE and TA, inversely) to adult eGFR and in large part also strongly significant to CL_Cr_ after adjusting for childhood and adulthood covariates and confounders relevant to kidney health and dietary intakes. This and the detected higher albuminuria after long-term higher renal ammonium excretion in otherwise healthy participants strongly suggest that in young years, regularly increased acid loads to the body already begin to adversely influence kidney function. Hence, as an important kidney health prevention measure, habitual consumption of diets with low potential renal acid loads that include appropriate amounts of alkalizing fruits and vegetables with clear ammoniagenic and NAE-lowering efficiency should be started early in childhood.

## Supplementary Materials

Supplementary File (PDF) Figures S1, S2; Item S1.
