# Supplementary material for: Renal Net Acid Excretion During Growth and eGFR, Creatinine Clearance, and Albuminuria in Young Adulthood
Source: Kidney Med. 2025 Dec 2;8(2):101195. doi: 10.1016/j.xkme.2025.101195 (PMC12830164; doi:10.1016/j.xkme.2025.101195)
Supplement: Supplementary File (PDF) — Figures S1, S2; Item S1. [file mmc1.pdf]

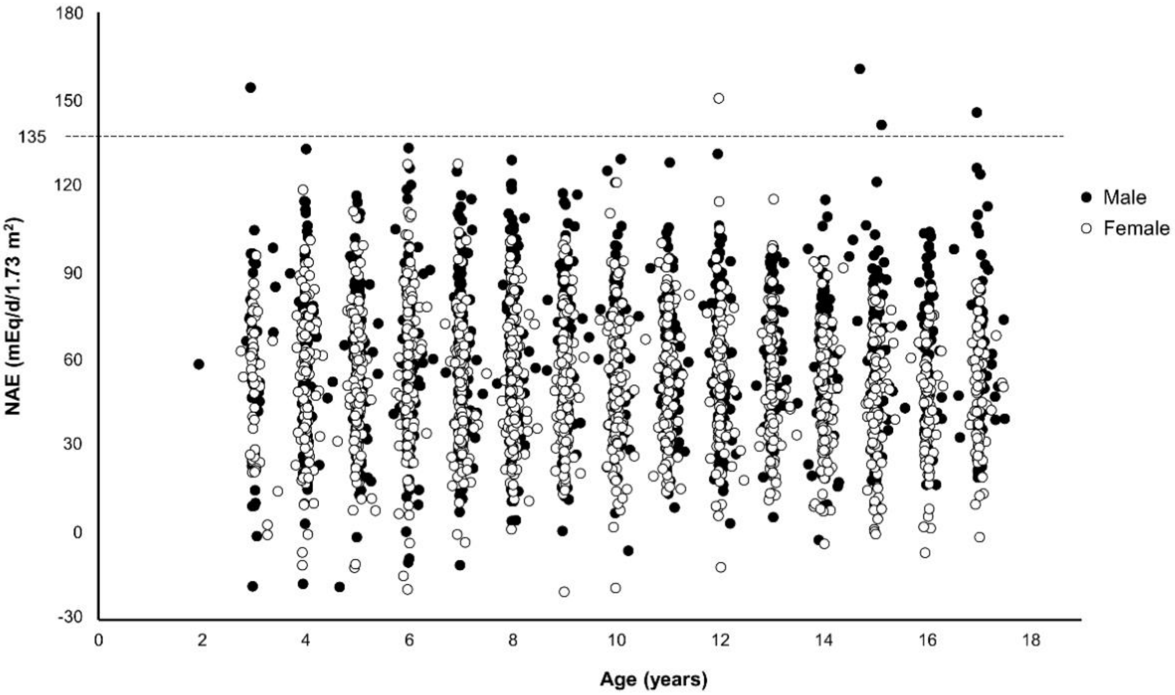

Figure S1. Scatterplot showing children´s and adolescents´ body surface corrected net acid excretion (NAE) depending on age (177 boys, 1747 twenty-four-hour urine samples; 177 girls, 1713 twenty-four-hour urine samples). The dotted line (135 mEq/d/1.73 m2) indicates the 24-h net acid excretion of healthy subjects going along with a 24-h urine pH of around 5.5.[4]

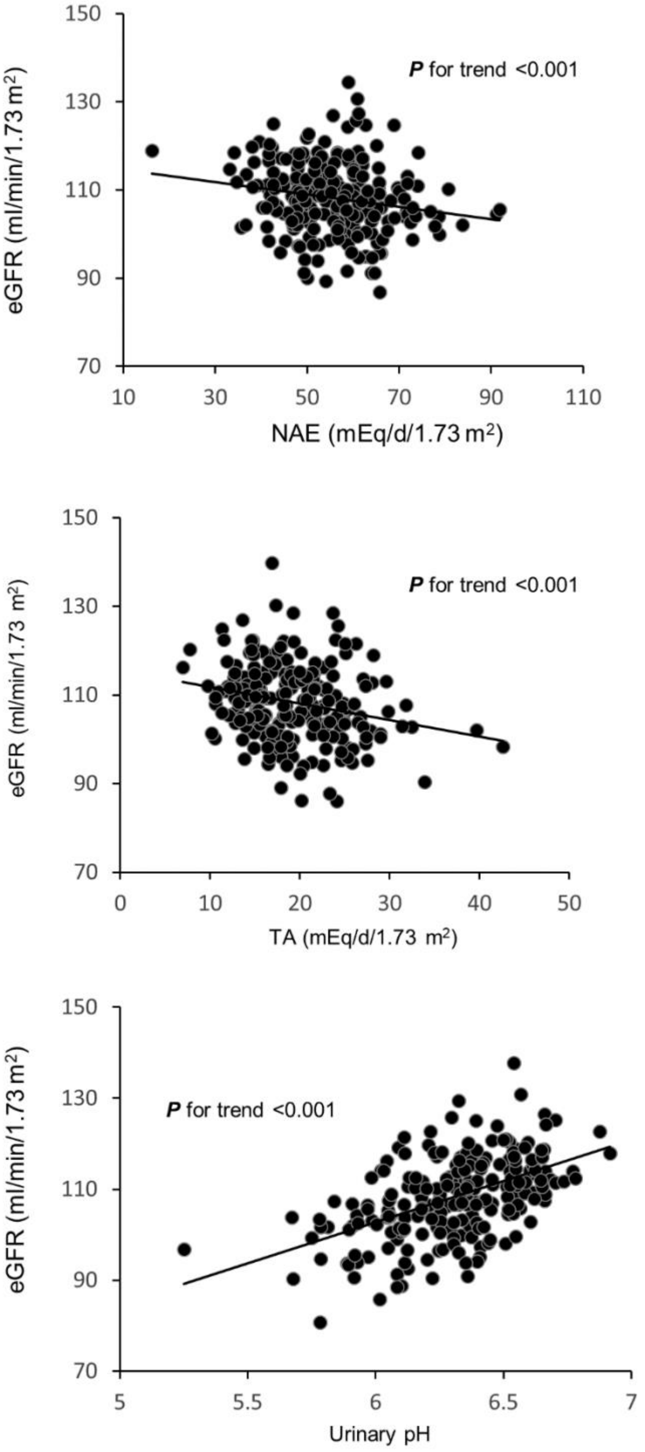

Figure S2. Sensitivity Analysis additionally allowing for the biomarker of protein intake, i.e., 24-h urinary urea nitrogen excretion in adulthood: Shown are prospective relationships of individuals’ means of body surface area-corrected NAE and titratable acidity and 24-h urine pH during pre-adulthood with eGFR in adulthood for all subjects with available 24-h urine collections in adulthood (n=216). Predicted values of eGFR were derived from multiple linear

regressions after adjustment for sex and 24-h urinary excretions of urea-nitrogen, creatinine, and potassium during growth. Additional inclusion of adult 24-h urinary urea nitrogen excretion and adult fat free mass (FFM) did not change the models’ R^2 or the predictors’ β value relevantly and were thus not considered. Significance levels for adult urea nitrogen included in the regressions, were $P > 0.18$ for all 3 predictors.

Item S1: Formulas Used in Methods

Calculation formulas for body mass index (BMI), body surface area (BSA) [1], adult fat free mass (FFM) [2], creatinine clearance (CrCl) and estimated glomerular filtration rate (eGFR) [3].

BMI [kg/m²] = weight/height²

$BSA\ [m^2] = 0.007184 * height\ (cm)^{0.725} * weight\ (kg)^{0.425}$

$FFM_{male}{}^{a} = 9.27 * 10^3 * body\ weight / (6.68 * 10^3) + (216 * BMI)$
$FFM_{female}{}^{a} = 9.27 * 10^3 * body\ weight / (8.78 * 10^3) + (244 * BMI)$

$eGFR_{male}\ [mL/min]^{b} = 142 * (sCr/0.9)^{y} * 0.9938^{Age}$
$eGFR_{female}\ [mL/min]^{c} = 142 * (sCr/0.7)^{z} * 0.9938^{Age} * 1.012$

$CrCl\ [mL/min] = Cr_{urine}\ [mmol/L] * Vol_{urine}\ [mL] / Cr_{serum}\ [mmol/L] * time_{urine\text{-}collection}\ [min]$

---

[a] Male and female FFM calculations are based on semi-mechanistic models, developed to predict lean body weight of adult subjects with a wide range of body weights[2].
[b] If serum creatinine (sCr) ≤ 0.9 then 'y' equals -0.302; if sCr >0.9, 'y' equals -1.200.
[c] If serum creatinine (sCr) ≤ 0.7 then 'z' equals -0.241; if sCr >0.7, 'z' equals -1.200.

Supplementary References

1. Du Bois D, Du Bois EF. A formula to estimate the approximate surface area if height and weight be known. 1916. *Nutrition*. 1989;5(5):303-11; discussion 312-3. (Reference 17 in manuscript)

2. Janmahasatian S, Duffull SB, Ash S, Ward LC, Byrne NM, Green B. Quantification of lean bodyweight. *Clin Pharmacokinet*. 2005;44(10):1051-1065. doi:10.2165/00003088-200544100-00004 (Reference 18 in manuscript)

3. Inker LA, Eneanya ND, Coresh J, et al. New Creatinine- and Cystatin C-Based Equations to Estimate GFR without Race. *N Engl J Med*. 2021;385(19):1737-1749. doi:10.1056/NEJMoa2102953 (Reference 18 in manuscript)

4. Remer T, Manz F. Estimation of the renal net acid excretion by adults consuming diets containing variable amounts of protein. *Am J Clin Nutr*. 1994;59(6):1356-1361. doi:10.1093/ajcn/59.6.1356 (Reference 23 in manuscript)
